# Shifting Horizons: The Impact of Global Events on the Intention to Migrate of the Next Generation Romanian Nurses

**DOI:** 10.3390/healthcare12060675

**Published:** 2024-03-17

**Authors:** Codruța Alina Popescu, Veronica Junjan, Anca Dana Buzoianu, Mugur Daniel Ciumăgeanu, Șoimița Mihaela Suciu

**Affiliations:** 1 Department of Human Science, University of Medicine and Pharmacy “Iuliu Hațieganu”, 400012 Cluj-Napoca, Romania; cpopescu@umfcluj.ro; 2 Section of Public Administration, Faculty of Behavioral Management and Social Sciences, University of Twente, 7522 NB Enschede, The Netherlands; v.junjan@utwente.nl; 3 Department of Pharmacology, Toxicology and Clinical Pharmacology, University of Medicine and Pharmacy “Iuliu Hațieganu”, 400012 Cluj-Napoca, Romania; 4 Department of Psychology, Faculty of Sociology-Psychology, West University, 300223 Timișoara, Romania; mugur.ciumageanu@e-uvt.ro; 5 Department of Physiology, University of Medicine and Pharmacy “Iuliu Hațieganu”, 400012 Cluj-Napoca, Romania; mihaela.suciu@umfcluj.ro

**Keywords:** migration, nursing students, Brexit, COVID-19 pandemic

## Abstract

Background: This article investigates the determinants of the intention to migrate of nursing students at a major medical university in Romania and relates them to major international developments, specifically the Brexit referendum and the COVID-19 pandemic. Methods: An online survey about the intention to migrate was made available to nursing students at the University of Medicine and Pharmacy “Iuliu Hațieganu”, Cluj-Napoca, Romania, in 2016 (before Brexit) and again in 2016 (after Brexit), 2017, 2018, and 2021 and 2022 (during the pandemic). A total of 549 students responded (response rate: 84.6%). Results: Before the Brexit referendum, 62.6% of the respondents had a plan to seek employment abroad, whereas after the Brexit referendum, only 34.7% indicated that they had such a plan after graduation. Before the pandemic, 43.6% of the students expressed an intention to work abroad, while during the pandemic, only 19.8% had such plans. Conclusions: This study documented the effect of significant international developments—such as the Brexit referendum and the COVID-19 pandemic—on decreasing the intention to migrate. As expected, the change in preference for the UK as a destination country changed dramatically. Additionally, the study provides both theoretical and empirical insights into the types of and the consistency of preparation for migration of nursing students.

## 1. Introduction

The migration of medical staff represents an increasing issue for both departing and receiving countries around the world. The medical brain drain has negative consequences in the form of under-provision of medical services in departure countries, who spend resources on the education and training of medical personnel and do not eventually reap the benefits of such investments. Receiving countries, in turn, also need to invest in the integration and adjustment of the newly arrived personnel, who are working in different cultural and professional settings. There is a broad body of research documenting the migration and adaptation issues that are faced by health personnel; here, we especially focused on nursing professions [1,2,3,4,5]. A rather peculiar case is the European Union (EU), where internal migration is replaced by the phenomenon of “mobility”, creating a paradoxical tension [1] between member states on the west–east coordinates, with governments being powerless in endorsing the recommendations of the WHO’s Global Code of Practice on the International Recruitment of Health Personnel [6].

The COVID-19 pandemic has triggered a significant upheaval in the global healthcare workforce, referred to as a “migration tsunami”. This crisis has placed immense pressure on healthcare systems worldwide, resulting in a critical shortage of healthcare professionals, especially in certain countries [7]. The pandemic not only highlighted, but also intensified, the already existing global demand for nurses. As international migration continues to increase, hospitals and healthcare organizations are actively seeking to augment their staff by recruiting nurses from other regions, especially in receiving countries like the USA [8], with the private sector (and sometimes also the public one) often ignoring or reinterpreting the WHO recommendations that are mentioned in the Global Code of Practice. On the other hand, before the pandemic, Brexit created disruptions to European Union migrant nurse recruitment and retention in the United Kingdom, with migrant nurses, a highly mobile and skilled workforce, feeling increasingly disenfranchised and insecure in their employment, mainly due to the post-Brexit citizen status and work regulations for EU nurses, but also due to xenophobic and racist sentiments [9].

In order to plan the training and employment of healthcare labor forces, it is essential that governing bodies are informed about in-training nurses’ intention to migrate and perceptions of their future career [10]. The recent literature has drawn attention to the issue of the intention to migrate and preferences for work locations of medical and nursing students, both of graduates of nursing studies inside and outside of EU borders [11,12,13,14,15]. Identifying the intention to migrate and understanding the reasons for migration among nursing students who are preparing for the future provide a first step to developing policy responses that adequately address the complex phenomenon of nurse migration. At the individual level, the decision to migrate has consequences for the person in terms of being away from family, friends, and their known environment. These consequences are combined with the pressures to build a new life in a foreign environment [16].

The migration of medical personnel is also an issue at the country level. On the one hand, the migration of medical personnel puts pressure on the departure countries, who invested in the education and training of the medical personnel and ultimately find themselves dealing with personnel shortages and the under-provision of medical services. On the other hand, it is an issue for the receiving countries, where, besides the apparently easy solution of hiring already trained personnel and “saving” education costs, medical care providers have discovered that investments are needed to support the integration of the new personnel both at the hospital organization level and in terms of language, cultural habits, and expectations [9,16]. In some cases, if there are not effective measures for countering the effects of acculturation and local labor regulations, the human capital is endangered, and nurses end up eventually working under their qualification level or in precarious and dehumanizing conditions [5].

The consequences of the personnel migration within the EU should also be mentioned: the free circulation of persons is one of the four fundamental freedoms of the EU, but in the context of different demographic dynamics and healthcare provision needs, as well as the wage differentials across the EU, the general direction of the migration flux of medical personnel is from east to west [1]. Medical personnel have evolved as a dynamic and mobile workforce, and macro-social events such as economic crises, wars, and other events are arguably influencing the patterns of mobility of healthcare workers [17] and the work relationships between and tasks of domestically trained nurses and foreign (but EU)-trained ones [18].

The current article investigates the determinants of the intention to migrate of first-year nursing students in a major medical university in Romania and follows their association to major international events, such as the British referendum on leaving the EU and the COVID-19 pandemic. After introducing the research problem and its background, this article presents an overview of the case of nursing education in Romania. Next, the description of a general model of professional migration based on push-and-pull factors is provided, followed by the description of the research method that is applied in this research project. The final section reports the results, a discussion of the results in light of the theoretical framework, some limitations and future directions of inquiry of the phenomenon, and some concluding remarks.

**Nursing Education in Romania**

Nursing education in Romania after the Second WW consisted of 12 years of general education, followed by 2 to 3 years of nursing education. In 1976 and the following years, in the middle of an obscurantist and anti-intellectual climate, the Ceaușescu regime closed schools of nursing, psychology, and social work [19,20]. Between 1976 and 1989, the education of nurses was carried out in vocational, secondary education level nursing schools. Between 1990 and 1995, two different training schemes in nursing education coexisted: secondary education and nursing colleges. Since 1995, nurses have been trained exclusively in nursing colleges (three years of study after the completion of high school) [2].

The adoption of the Bologna criteria and the necessity to implement EU requirements (Directive 2005/36/EC) in nursing training led to significant changes in the organization of nursing education [21]. In 2003, in compliance with EU accession requirements, nursing education moved to programs that are offered at the university level, with four-year bachelor level undergraduate programs (organized to provide 240 ECTS credits).

Despite these EU accession requirements, Law 307/2004 [22] allowed private nursing colleges to offer non-degree training programs and provided their existing students with the chance to finalize their studies, even though after 2007, these colleges should not have enrolled new students. In 2008, the Romanian government obtained permission from the EU to continue nursing training in colleges and for these diplomas to be recognized at the EU level [23], but stronger accreditation and quality control requirements were introduced for these schools [2]. A total of eleven medical schools in the country offer professional nursing programs at the university level. According to public data on the universities’ web sites, there were a total of 1145 places available in 2017. Although these courses are based in public universities, most students self-finance their education due to the limited number of tuition-free slots (usually about one-third of the total number of available slots). In 2017, the cost per year was the equivalent in national currency of EUR 900. Admission to public nursing schools is usually based on a multiple-choice test on anatomy.

**Theoretical Model and Aim of the Study**

The literature discusses the factors influencing the decision to migrate extensively [4,24]. Particularly relevant for the current article are the developments regarding the increased migration of medical personnel at the macro-, meso-, and micro-/individual level. From a general theoretical perspective, medical and nursing migration (with nursing migration being especially relevant for the present paper) is rather puzzling in the present-day EU context. Classic economic theories related to migration do not apply very well—neo-classical theories based on Lewis’ framework of analysis of the post-colonial contexts related to economic development with unlimited supplies of labor [25] apply only in part to the reading of the contemporary migration phenomenon. Rather, the dual-economy migration model, based on Lewis’ framework, was rejected by the change in migration features and ideologies since the mid-1970s, where more sociological or even mathematical behavioral prediction models [26] were formulated.

Sociological theories fit better for the present issues, even if the classic push–pull factor theory [27] is also well outdated, or at best replaced by more contemporary variants, such as the transnational social spaces model [28], or by mixed-model theories, which combine both economical and sociological outlooks on the issue (such as the world system theory or the migration systems theory, found in [29], p. 5). To provide a more nuanced interpretation framework for the present paper, the approach that we apply brings in additional psychological perspectives at the personal level on the migration process, such as the migration change model [30] or resource-based models based on Berry’s general acculturation framework and Hobfoll’s conservation of resources stress theory [31].

Such a broad theoretical framework allows us to analyze the process of forming an intention to migrate at different levels, keeping both socioeconomic and psychological features in mind. At the macro-economic and policy levels, the public sector reforms of recent decades had profound consequences for the way in which health systems coped with the planning for and education of sufficient personnel, both in developing and developed countries. Limits in public spending have led to budget shortages and hiring freezes, which resulted in increased migration of medical staff from developing countries, for whom OECD countries became particularly attractive destinations [32].

Kingma [4] provides a very clear overview of the change in direction of the migration of nurses from developing towards developed countries, with relevant nuances on the “two-step” approach where nurses use intermediary stops to develop their skills and abilities to work in international contexts, which help their eventual applications for work in OECD countries. Macro-level reforms had consequences at the meso-level (organizational level), where hospitals had to cope with extra monitoring and administrative tasks, which increased the workload and time pressure for medical personnel and conflicted with their direct-medical-care tasks, leading, in some cases, to the departure of educated personnel for the private sector or for other fields of work.

At the individual level, factors such as age, family ties, employment opportunities, and existing cultural similarities do play a role in the decision to migrate [33]. There are also barriers to migration which need to be taken into account: the physical costs of moving, professional accreditation, learning a new language and the professional terminology in a new language, learning new clinical practices, and learning to address patients’ needs being expressed with a different set of nonverbal and cultural cues [34,35].

The aim of the present descriptive study was to investigate the evolution across time of the intention and preparation to migrate for work of Romanian nursing students by answering the following research questions:

1. How do the intentions of Romanian nursing students regarding migration vary over time?
2. Which push factors drive Romanian nursing students to consider migration?
3. Where do Romanian nursing students intend to migrate to?
4. How long do Romanian nursing students plan to stay abroad?
5. What pull factors and obstacles do Romanian nursing students face when deciding to migrate?
6. To what extent are unexpected international events such as Brexit and the pandemic associated with variations in the migration decisions of Romanian nursing students, and what are their preferred destination countries?

## 2. Materials and Methods

This study employed a cross-sectional, observational, questionnaire-based data collection process, conducted at the University of Medicine and Pharmacy “Iuliu Hațieganu” Cluj-Napoca in February 2016, November 2016, November 2017, November 2018, January 2021, and November 2022. Students from the first-year nursing section were recruited, and our focus on the positions expressed by the students in their first year allowed us to assess the evolution of the baseline of their intention and preparation to migrate for work. Of the 650 Romanian nursing students who were approached during the 6 periods of the study, 549 agreed to participate (response rate: 84.46%). From the second wave of the survey, we included a specific item related to the possible changes in the students’ immigration plans associated with the outcome of the Brexit referendum, and for the surveys in 2021 and 2022, a question about the possible changes in their immigration plans associated with the COVID-19 pandemic was included.

### 2.1. Procedure

This study was conducted in close collaboration with the nursing students’ representatives. An electronic version of the survey was made available to all students in nursing through their closed communication platforms. Participation in the survey was voluntary, and the students did not receive any incentives to participate in the study. The study received approval from the Research Ethics Committee of the UMPh “Iuliu Hatieganu” Cluj-Napoca (approval number 104/2016).

### 2.2. Survey Instrument

Based on international publications and surveys about nurse migration [4], the authors developed a 50-question survey questionnaire focused on the push–pull theory, which is used to explain the circumstances of nurse migration. The same questionnaire, without the added questions on the pandemic and Brexit, was also used in a previously published study about the migration intent of medical students [36].

For the development of the questionnaire (the Medical Migration Push and Pull Factors Questionnaire), the authors used the push and pull theoretical framework [27], enriched with some elements based on the acculturation framework and social spaces model [28,31]. Below is a description of the questionnaire’s development phases:

Phase 1: Item construction

Items were developed based on a literature review, and extra items were extracted by means of two focus groups: one with managers and heads of departments of hospitals who deal with the migration of healthcare personnel, and one with medical and nursing staff who have migration experience. The literature review was conducted searching PUBMED for relevant articles using the search terms “Migration intent of medical students” and “Migration intent questionnaire, nursing students”, and 12 papers were considered relevant for the topic and were reviewed.

Phase 2: Item screening

Items were screened using the Delphi technique (two-round expert consultation). Twenty experts were recruited according to the following inclusion criteria: experience with the migration of nurses (healthcare managers, managers of recruitments firms who facilitate migration), nurses with experience of working abroad, and nursing students who have undertaken summer internships in a foreign country. The Delphi inquiry was conducted via e-mail. In round one, the experts were asked to rate the items on a five-point Likert scale (0 to 4, where 0 = strongly disagree and 4 = strongly agree), indicating to what extent they thought each item should be included in the questionnaire. After round one, the central tendency was evaluated by item selection rates (the number of experts who rated items with a score of 3 = agree, or 4 = strongly agree/the total number of experts × 100%). Items that scored less than 80% were deleted. The amended questionnaire was assessed by the same expert panel in round two. The experts were asked to re-rate the amended items on the same five-point Likert scale. The central tendencies and discrete degree were computed. The discrete degree was measured using the coefficient of variation (the ratio of the standard deviation and arithmetic average of an item’s importance score). Items with an item selection rate < 80% and an item coefficient of variation > 0.2 were deleted.

Phase 3: Questionnaire validation

The questionnaire was distributed to nursing and medical students. In total, 120 students answered the questionnaire, and the data were used for the validation. The internal consistency was assessed by calculating Cronbach’s alpha coefficient. Cronbach’s alpha coefficient was 0.793 for the questionnaire, 0.83 for the push sub-scale, and 0.764 for the pull sub-scale. The construct validity was assessed by means of principal component analysis (PCA). PCA is often used in the context of questionnaire validation to explore the underlying structure of the data and to identify any patterns or relationships among the variables. PCA can help to identify underlying dimensions or factors in the data by reducing a large number of variables to a smaller set of principal components. This can simplify the analysis and interpretation of the data. If the items load strongly on the expected components and the structure of the components aligns with theoretical expectations, this provides support for the validity of the questionnaire. Bartlett’s test of sphericity, which assesses the equality of variance in different populations, and the Kaiser–Meyer–Olkin (KMO) test, which is a measure of the sampling adequacy, were conducted to confirm the suitability of data. To determine the structure of the underlying factors of the questionnaire, the eigenvalue > 1 and the scree plot of eigenvalues were determined. The number of factors to be extracted was determined by inspecting the scree plot. A *p*-value < 0.05 was considered statistically significant. The KMO value was 0.804, and the result of Bartlett’s test was statistically significant (*p* < 0.000), which indicated that the data were suitable for factor analysis. The PCA identified two factors when loading the results, the push factor and the pull factor, which accounted for 86.742 item variances.

The final survey instrument (available on request) consists of four parts. The first part contains questions concerning the sociodemographic status of the students, their prior personal migration experience, and family members working abroad. The second part contains questions related to their migration decision, push and pull factors for migration, and concrete departure steps (enrollment in language courses, attending job fairs, searching jobs on the Internet, and talking with Romanian nurses working abroad). The third part contains questions about their satisfaction with their future working conditions and earnings in Romania and abroad and about discrimination at work. The responses were recorded by using five-point Likert scales (answers ranging from never to always, dissatisfied to satisfied, and unlikely to very likely). The fourth part contains questions about the ethics of migration and freedom of movement.

The questionnaire was administered to students in February 2016, November 2016, 2017, and 2018; January 2021; and November 2022. They also included three questions about students’ perception of the impact of the Brexit referendum on their migration decision, and in January 2021 and November 2022, a question about the perceived impact of the COVID-19 pandemic was included.

### 2.3. Statistical Analysis

We first conducted a descriptive analysis of the students’ demographic characteristics and of the outcome variables of interest (migration intention, destination country), using mean and standard deviation for continuous variables and frequencies and percentages for categorical variables. The normality of the distributions of the data was first assessed using Shapiro–Wilk’s tests. Because the data were not normally distributed, between-group comparisons of the continuous variables were performed using the Mann–Whitney U-test. Multiple linear regression was used to predict nursing students’ intent to work abroad. The results were considered statistically significant if the *p*-value was lower than 0.05. Multiple linear regression is a suitable statistical technique when the goal is to predict a continuous outcome variable based on multiple predictor variables. Since there are multiple predictors involved, each potentially contributing to the outcome, multiple linear regression allows for the examination of the unique contribution of each predictor while controlling for the effects of other predictors. The categorical variables with more than two categories were appropriately handled by recoding and dichotomizing them. This simplifies the analysis while retaining the essential information that is encoded in these variables. Analyses were run using SPSS version 24 for Windows 11.

## 3. Results

A total of 549 students were included in this study. Table 1 shows the sociodemographic characteristics of the participants, with data expressed as the mean ± standard deviation, numerically, and as percentages.

The students’ ages ranged from 18 to 47 years, with a mean age of 19.81 (±3.15) years. The students’ assessment of their household’s economic situation was that 4.4% were living in precarious conditions, 20.6% stated that they cannot afford everything that is needed for a normal life, 68.5% indicated that can afford everything that is needed for a normal life, and 6.6% said that can consume without any restrictions.

Most of the students’ parents (57.4% of mothers and 63% of fathers) had completed high school. Secondary school graduation was achieved by 17.7% of mothers and 17.9% of fathers. Additionally, 24.8% of mothers and 19.1% of fathers held a university degree, and 8.7% of the students’ parents were nurses.

We ascertained that 3.1% of students worked part-time, and 2.9% worked full-time. In addition, 89.4% of students spoke a foreign language, and 53.4% spoke two foreign languages. The most spoken language was English (73.2%), followed by Hungarian (43%) and French (40.1%).

While 47% of the students were paying a tuition fee, for 53% of the students, their education was free.

### 3.1. Desire to Emigrate and Destination Country

Table 2 shows the distribution of the students’ intention and willingness to work abroad.

Before the Brexit referendum, 62.6% of the respondents had a reasonably developed plan to seek employment abroad after graduation, whereas after the Brexit referendum, only 34.7% indicated that they had such a plan. Before the pandemic, 43.6% of the students expressed an intention of working abroad, while during (and after) the pandemic, only 19.8% had such plans.

Table 3 presents the preferred destination countries of the students.

Among the students who were planning to emigrate, the preferred host countries were Great Britain (26.4% before the Brexit referendum and 14.8% after), Germany (9.9% before the Brexit referendum and 10.4% after), Ireland (0% before Brexit, 2.1% after Brexit), and the United States (4.4% before Brexit and 2.7% after). Roughly 29.5% of the students did not know (at the moment of answering the questionnaire) which country they wanted to emigrate to.

### 3.2. Timeframe for Migration and Preferred Length of Stay in Host Country

Table 4 presents the students’ preferred length of stay in the host country.

Before the Brexit referendum, most students wanted to go abroad for a short period: 17.6% planned to stay abroad a few months each year, 46.2% wanted to stay abroad for several years and then come back to practice in Romania, and 17.6% of the students had a permanent emigration plan (see Table 4). After the Brexit referendum, the students still expressed a preference for short-term rather than permanent migration, as 14.4% planned to stay abroad a few months, 28.2% wanted to stay abroad for several years and then come back to practice in Romania, and 11.4% of the students had a permanent emigration plan. However, the percentage of students who wanted to stay in Romania increased from 18.7% to 46.1%. Comparing the pre-pandemic and pandemic periods, the proportion of students who wanted to emigrate permanently decreased from 14.9% to 8.2%.

Table 5 presents the students’ planned timeframe for migration.

Before Brexit, 22% of the students planned to leave after graduation, 59.3% intended to leave after working a few years in Romania, and only 18.7% of the students did not have any plans for migration (see Table 3). After Brexit, 13.8% of the students planned to leave after graduation, 40.2% intended to leave after working a few years in Romania, and 46.1% of the students did not have any plans for migration. After the pandemic, the percentage of students who wanted to leave immediately after graduation decreased.

### 3.3. The Factors Influencing the Decision to Migrate

This section provides an account of the main factors that influence the decision to work abroad. These factors could be potential subjects of inquiry for health policy makers if they are trying to reduce Romanian nurses’ intention to migrate. The respondents were asked to rank the following reasons for leaving the country on a 0 (completely disagree)–100 (completely agree) scale: “higher wage abroad”; “better living conditions abroad”; “I am disappointed with the Romanian health care system”; “to gain living and working experience abroad”; “personal reason (my partner wants to work/is working abroad)”; “better professional opportunities”; and “lack of working places in Romania” (see Table 6). Respondents were also asked to rank the following reasons for staying in Romania on a 0–100 scale: “patriotism”, “family is in Romania”, “friends are in Romania”, and “it is more difficult to work abroad”.

For push and pull factors, we also included a question about anticipated discrimination abroad (Table 7).

The results in Table 7 indicate that there was no difference in the students’ expectation of discrimination abroad between students who wanted to emigrate and the ones who did not want to emigrate.

### 3.4. Concrete Departure Preparation and Planning

The students who planned to emigrate had usually taken concrete steps towards their emigration goals, including enrolling in language courses (12.6%), searching for jobs on the Internet (33.7%), and contacting Romanian nurses who work abroad (57.9%). There were significant differences in the level of preparation between the students who planned to emigrate and the students who did not plan to leave Romania (see Table 8), and although our students were in their first year and had four years to finish their nursing degree, the students who had the desire to leave the country had already begun preparing for working abroad.

There was a statistically significant difference between the students who were intending to emigrate and the ones who were not in terms of the push and pull factors.

Two multiple linear regressions were calculated to predict the push and pull factor scores based on the socioeconomic and demographic variables (gender, financial situation, marital status, residence, financing of studies, satisfaction with potential salary after graduation, knowing one or two foreign languages, and having a family member abroad). Categorical variables with more than two categories were recoded and dichotomized (financial situation, marital status). All predictor variables were entered simultaneously.

An exploratory multiple regression analysis examined the predictors of push factors (see Table 9).

Having a family member abroad (*p* = 0.000), living in a rural area (*p* = 0.02), and being unsatisfied with the potential salary as a nurse in Romania (*p* = 0.000) significantly predicted higher scores for push factors. All other predictors were unrelated to the push factors (all *p* > 0.05). The overall model fit was R^2^ = 0.81 (adjusted R^2^ = 0.65).

An exploratory variable multiple regression analysis examined the predictors of pull factors (see Table 10).

Having a family member abroad (*p* = 0.001), being unsatisfied with the potential income in Romania (*p* = 0.02), being unsatisfied with the potential salary as a nurse in Romania (*p* = 0.014), and being in a lower financial situation (*p* = 0.001) predicted higher negative scores/being more likely to search for employment abroad, i.e., higher scores for pull factors. All other predictors were unrelated to the pull factors (all *p* > 0.05). The overall model fit was R^2^ = 0.64 (adjusted R^2^ = 0.49). Students who did not have a family member abroad, were satisfied with the potential income, and had a good financial situation were less likely to search for employment abroad.

### 3.5. The Impact of Brexit and the Pandemic on Emigration Intention

Table 11 presents the impact of Brexit on emigration intention.

The outcome of the Brexit referendum seems to be associated with the first-year nursing students’ intention to migrate, but still, 25% of the students expressed the desire to work in the UK.

We asked the students (in the survey in 2021 and 2021) if the pandemic had an impact on their desire to emigrate. While 55.1% of the students did not want to leave, 33.3% did not indicate that the pandemic had an impact on their plans, and 11.6% still wanted to emigrate but postponed their decision.

### 3.6. Ethics and Migration of Healthcare Workers

Because of the shortage of nurses and doctors in Romania, there is a debate in the media about introducing a tax on the migration of highly skilled medical personnel. One such proposal argues for introducing a compulsory initial period of working in the country for doctors and nurses for several years after their graduation or that they must reimburse the cost of their education if they migrate. We asked the nursing students how they felt about this policy proposal. Only 34.4% of the students agreed with the idea that nurses who wish to leave the country should work for a few years in Romania, and that if they do not, they should reimburse the cost of their education.

## 4. Limitations and Discussions

This study aimed to investigate the determinants of the intention of first-year nursing students in a major medical university in Romania to migrate and examined their association with major international events. The current study’s contribution is twofold: to understand the determinants of the decision to migrate and the preparation for migration of nursing students, and to provide a plausible interpretation of the impact of international developments (like Brexit or the COVID-19 pandemic) in the students’ preparation for migration and in choosing their preferred destination countries.

Before summarizing and discussing the study’s results, it is worth mentioning some of its limitations. Firstly, our analysis focuses solely on the migration intent of nursing students within the initial year, precluding generalizations about migration intentions at later stages of studying or the actual migration outcomes. Additionally, the study’s timeframe, spanning from 2016 to 2022, presents another constraint. Despite numerous significant international events occurring during this period, it is impractical to include them all in our analysis. Instead, we have chosen to specifically examine the association of the intention to migrate with events such as Brexit, which affect the students’ potential right to work abroad, as well as factors like border closures during the pandemic, which influence their freedom of travel and the potential for familial separation (a significant number of the students had family abroad).

Another limitation of this study is that even though the design is a six-year longitudinal study of first-year nursing students, the number of participants is relatively small and unevenly distributed along the assessment waves. The study relies solely on survey questions, and although the questionnaire has sound psychometric properties, the use of supplementary scales for measuring the personality factors or work-related features of participants would have been beneficial (for example, the use of measures related to career adaptability and optimism—see [10,37]; or measures related to the acculturation process and a predicted outlook of the individual related to assimilation, separation, integration, or marginalization processes—see the bidimensional directions of assessment in [38]). A follow-up of the consequences and practicalities of emigration in the context of intra-EU mobility could be beneficial—following the migrant nurses in the host countries could provide data about the discrepancies between expectations and the possibly difficult adaptation to a new environment (taking acculturation processes into account).

It is estimated that around 2.7 million Romanian migrants currently live in another European country, and even if this trend of migration is now slowing down, it is still notable and a potentially human-capital-draining process [39]. For these migrants, at the beginning of the migration process at least, labor activities abroad led to an improvement in wages and occupation in comparison to their preceding job in their home country. Parents who are working abroad finance the education of their children in Romania, and with this, they increase the skill levels of people in the country. This should be, in principle, a positive effect of the migration, but our study showed that students who have family abroad are more likely to express a desire to emigrate.

Regarding this finding, the first notable result comes from the regression analysis. Among nursing students, having family abroad, not being satisfied with the potential income in Romania, and living in rural areas were predictors of emigration intention. It is interesting to note that gender or one’s own economic situation were not significant factors impacting the intention to migrate. This combination of factors suggests that workforce migration has entered a second phase, where an already established (family) network and language skills are considered more important [34]. Better fitted to a theoretical outlook are, therefore, not a neo-classical dual-economy model or a clearcut push–pull factor model, but a translational social spaces model [28] or a migration change model [30], which imagines the future migrant as more agentic and aware of the acculturation process, rather than simply an individual looking for a higher economic status, regardless of their working conditions. From this point of view, 73.2% of nursing students estimated that they knew English at a B2 level, and proficiency in the host country’s language influenced nurses’ migration decisions, which concurs with the factors facilitating the integration of new personnel [31].

We found that 43.5% of the nursing students had a family member living abroad, and other studies have shown that having family living abroad is also an important pull factor regarding emigration intentions [13,33]. The household’s economic situation was not a prediction for emigration intent in the nursing students, a result that is different from a study that was carried out in Serbia [13], but that is similar to results from a Lithuanian sample of students [40]. Also, the vast majority of the students do not have any income and are supported by their parents, so their view of the economic situation of the household may not be realistic. There are numerous studies that have shown that emigration intention is stronger in men than in women [35,41,42]. We did not find significant differences in our sample, but this could be due to the skewed gender distribution in our respondents, where the majority (91.6%) of our students were women.

Regarding macro-influences, with Brexit and the pandemic being the main suspects in this context, the figures that we extracted from our study are in line with EU or international trends. Mckee [43] showed that between July 2016 and April 2017, there was a drop of 96% in nurses from the EU applying to join the workforce in the EU, so the decrease in the emigration intent of Romanian nursing students follows this tendency, as the UK was at the main destination country at the beginning of this study. Before Brexit, 62.6% of the respondents estimated a rather high likelihood of emigrating, whereas after Brexit, only 29% had such plans. Before the Brexit referendum, the number of nursing students who intended to work abroad was very high, similar to that of Serbian nursing graduates (69.7%) [13] or Korean nursing students (69.8%) [44]. After Brexit, the number was lower than the 40% of Polish nursing students who intended to emigrate [45]. The COVID-19 pandemic further decreased the percentage of students who were highly likely to emigrate, as only 19.8% of the students in our study had such plans.

Regarding repatriation intention, before Brexit, 17.6% of the students had a permanent migration plan, whereas the number dropped to 11.4% after the referendum. Most students only intended to leave Romania temporarily, either for a few months (17.6% before, 14.4% after Brexit) or for several years (46.2% before, 38.2% after Brexit). Comparing the pre-pandemic and pandemic period, the students who wanted to emigrate permanently decreased from 14.9% to 8.2%.

Having a nursing degree is a potential pathway for emigration [46]. Is this the case for Romanian nurses? On the one hand, the state invests in the training and will hypothetically lose this investment due to migration. But there are also other factors that complicate this, and the trajectories are increasingly complicated in the present-day EU context [47], even before certain major events such as Brexit or the pandemic. In Romania, there is a disparity between doctors and nurses regarding the financing of their education. The majority of places for medical students (70–80%) are financed by the Ministry of Education, whereas for nursing students, only 30% are financed. Although the tuition cost per year is not prohibitive, even by local standards (EUR 967.74), the fact that the majority of nursing students are paying for their education potentially gives them a moratorium space that might eventually lead to contemplating migration. In this study, we asked nursing students what they think about the idea that a nurse who had their education paid for by the state should work in their home country for a few years, and that if not, they should reimburse the cost of their education. Only 34.4% of the students agreed with this proposal, a figure that is closely related to the number of state-funded places. Of course, if the government should propose such a measure for medical personnel only, it would be discriminatory, and subject to attack through juridical procedures. To the best of our knowledge, this is the first time that this type of question was asked in a study of persons who could be directly affected by such a measure.

## 5. Conclusions

This study documents the association of significant international developments—such as the Brexit referendum—with a decreasing intention to migrate amongst first-year nursing students in Romania. As expected, the preference for the UK as a destination country changed dramatically. Additionally, this study provides insights on the types and consistency of nursing students’ preparation for migration. Primarily, the presence of family abroad and a strong investment in one or two languages are significant in this respect. Finally, the study investigates the role of the perceived work conditions in the home country in the decision to migrate. As expected, a negative perception of the working conditions strengthens the motivation and the preparations to migrate. Further inquiries, both qualitive and quantitative, could shed more light on this phenomenon, especially if future work is centered on psychological factors affecting the decision to migrate.

## Figures and Tables

**Table 1:** Characteristics of the population/sample demographics (N = 549).

Gender (N, %)		
Male	40	7.3%
Female	509	92.7%
Age (years) (mean ± SD)		
	19.81	±3.11
Residence (N, %)		
Urban	367	66.8%
Rural	182	33.2%
Relationship status (N, %)		
Single	357	65%
In a relationship	144	26.2%
Married	48	8.8%
Economic situation of the household (N, %)		
Living in precarious conditions	24	4.4%
Can manage generally	113	20.6%
Can afford everything needed for a normal life	376	68.5%
Can consume without restriction	36	6.6%
Family abroad (N, %)		
No	310	56.5%
Yes	239	43.5%
Language skills at B2 level (N, %)		
One language	491	89.4%
Two languages	293	53.4%
Known languages		
English	422	76.86%
French	220	40.07%
Spanish	110	20.03%
German	62	11.29%
Italian	89	16.2%
Hungarian	236	42.98%

**Table 2:** Intention to work abroad.

		Before Brexit	After Brexit				
		February 2016	November 2016	November 2017	November 2018	January 2021	November 2022
Do not want to go	N	17	35	32	30	40	74
	%	18.70%	43.20%	37.20%	35.70%	48.80%	59.20%
Vague plan (<25%)	N	17	13	26	23	20	32
	%	18.70%	16.00%	30.20%	27.40%	24.40%	25.60%
Developed plan (25–50%)	N	17	19	10	15	12	3
	%	18.70%	23.50%	11.60%	17.90%	14.60%	2.40%
Definite plan (51–75%)	N	28	12	17	14	8	9
	%	30.80%	14.80%	19.80%	16.70%	9.80%	7.20%
Categorical plan (76–100%)	N	12	2	1	2	2	7
	%	13.20%	2.50%	1.20%	2.40%	2.40%	5.60%
**Overall**							
		**Before Brexit**		**After Brexit**		**χ^2^**	***p***
		**N**	**%**	**N**	**%**		
Do not want to emigrate (probability of leaving 0–25%)		34	37.4%	326	71%	37.87	0.000
Intend to emigrate (probability of leaving >26%)		57	52.6%	133	29%		
		**Before Pandemic (2016–2018)**		**During Pandemic**		**χ^2^**	***p***
		**N**	**%**	**N**	**%**		
Do not want to emigrate (probability of leaving 0–25%)		193	53.4%	166	80.2%	31.16	0.000
Intend to emigrate (probability of leaving >26%)		149	43.6%	41	19.8%		

**Table 3:** Preferred destination countries.

		Before Brexit	After Brexit
I don’t want to leave	N	17	163
	%	18.7%	48.4%
I don’t know	N	26	43
	%	28.6%	12.8%
France	N	1	3
	%	1.1%	0.9%
Germany	N	9	35
	%	9.9%	10.4%
UK	N	24	50
	%	26.4%	14.8%
Switzerland	N	1	3
	%	1.1%	0.9%
Sweden	N	0	2
	%	0.0%	0.6%
Belgium	N	0	7
	%	0.0%	2.1%
United States	N	4	9
	%	4.4%	2.7%
Australia	N	1	0
	%	1.1%	0.0%
Canada	N	0	2
	%	0.0%	0.6%
Hungary	N	1	2
	%	1.1%	0.6%
Norway	N	2	1
	%	2.2%	0.3%
Italy	N	1	2
	%	1.1%	0.6%
Spain	N	2	3
	%	2.2%	0.9%
Austria	N	2	2
	%	2.2%	0.6%
Denmark	N	0	3
	%	0.0%	0.9%
Ireland	N	0	7
	%	0.0%	2.1%

**Table 4:** The length of stay in the host country.

		February 2016	November 2016	November 2017	November 2018	January 2021	November 2022
I don’t plan to leave	N	17	35	32	30	40	74
	%	18.70%	43.20%	37.20%	35.70%	48.80%	59.20%
A few months each year	N	16	8	19	17	8	14
	%	17.60%	9.90%	22.10%	20.20%	9.80%	11.20%
A few years then I want to come back	N	42	26	26	23	26	28
	%	46.20%	32.10%	30.20%	27.40%	31.70%	22.40%
Permanently	N	16	12	9	14	8	9
	%	17.60%	14.80%	10.50%	16.70%	9.80%	7.20%
**Overall**							
		**Before Brexit (February 2016)**		**After Brexit**		**χ^2^**	***p***
		**N**	**%**	**N**	**%**		
I don’t plan to leave		17	18.7%	211	46.1%	24.48	0.000
A few months each year		16	17.6%	66	14.4%		
A few years then I want to come back		42	46.2%	129	28.2%		
Permanently		16	17.6%	52	11.4%		
		**Before Pandemic (2016–2018)**		**During Pandemic (2021–2022)**		**χ^2^**	***p***
		**N**	**%**	**N**	**%**		
I don’t plan to leave		114	33.3%	114	55.1%	26.20	0.000
A few months each year		60	17.5%	22	10.6%		
A few years then I want to come back		117	34.2	54	26.1%		
Permanently		51	14.9%	17	8.2%		

**Table 5:** Timeframe for migration.

			February 2016	November 2016	November 2017	November 2018	January 2021	November 2022
I don’t plan to leave		N	17	35	32	30	40	74
		%	18.70%	43.20%	37.20%	35.70%	48.80%	59.20%
I will leave as soon as I graduate		N	20	9	15	13	8	18
		%	22.00%	11.10%	17.40%	15.50%	9.80%	14.40%
I will leave after I work a few years in Romania		N	54	37	39	41	34	33
		%	59.30%	45.70%	45.30%	48.80%	41.50%	26.40%
**Overall**								
			**Before Brexit (February 2016)**		**After Brexit**		**χ^2^**	***p***
			**N**	**%**	**N**	**%**		
I don’t plan to leave			17	18.7%	211	46%	23.54	0.000
I will leave as soon as I graduate			20	22%	63	13.8%		
I will leave after I work a few years in Romania			54	59.3%	184	40.2%		
			**Before Pandemic (2016–2018)**		**During Pandemic (2021–2022)**		**χ^2^**	***p***
			**N**	**%**	**N**	**%**		
I don’t plan to leave			114	33.3%	114	55.1%	25.36	0.000
I will leave as soon as I graduate			57	16.7%	26	12.5%		
I will leave after I work a few years in Romania			171	50%	67	32.4%		

**Table 6:** Push and pull factors.

	No Intention to Emigrate (Probability of Leaving 0–25%) (N = 359)		Intention to Emigrate (Probability of Leaving >26%) (N = 190)		*p* (Mann–Whitney U)
	Mean	SD	Mean	SD	
**Push factors**					
higher wage abroad	60.4	36.27	83.55	18.64	0.000
better working conditions	62.8	35.01	87.62	16.92	0.000
disappointed with the Romanian healthcare system	52.3	31.43	71.47	26.33	0.000
better living experience abroad	36.9	32.54	71.99	28.43	0.000
my partner wants to work/is working abroad	17.1	28.95	24.85	37.70	>0.05
better professional opportunities	49.6	37.15	70.3	25.93	0.000
lack of working places in Romania	18.6	26.00	36.06	28.60	0.000
**Total score of push factors**	297.65	161.45	445.87	104.28	0.000
**Pull factors**					
patriotism	42.83	34.43	40.37	48.92	0.04
family is in Romania	79.44	31.23	65.46	32.90	0.000
friends are in Romania	65.54	34.77	56.87	30.89	0.000
it is more difficult to work abroad	46.99	31.45	39.54	27.81	0.005
**Total score of pull factors**	234.31	104.33	202.26	103.58	0.000

**Table 7:** Expected discrimination abroad.

How Likely Is It to Face Discrimination Abroad		No Intention to Emigrate (Probability of leaving 0–25%)	Intention to Emigrate (Probability of Leaving >26%)	χ^2^	*p*
not very likely	N	276	156	2.02	>0.05
	%	76.90%	82.10%		
very likely	N	83	34		
	%	23.10%	17.90%		

**Table 8:** Concrete departure preparations.

**Concrete Departure Preparation**		**Migration Intention**		**χ^2^**	***p***
		**No Intention to Emigrate (Probability of Leaving 0–25%)**	**Intention to Emigrate (Probability of Leaving >26%)**		
Job search on the Internet					
No	N	320	126	42.45	0.000
	%	89.10%	66.30%		
Yes	N	39	64		
	%	10.90%	33.70%		
Jobs fairs for healthcare professionals					
No	N	349	188	1.74	>0.05
	%	97.20%	98.90%		
Yes	N	10	2		
	%	2.80%	1.10%		
Talking with Romanian nurses who are working abroad					
No	N	232	80	25.68	0.000
	%	64.60%	42.10%		
Yes	N	127	110		
	%	35.40%	57.90%		
Enrolling in language courses					
No	N	342	166	11.21	0.001
	%	95.30%	87.40%		
Yes	N	17	24		
	%	4.70%	12.60%		

**Table 9:** Linear regression analysis for predictors of push factor score.

	Unstandardized Coefficients		Standardized Coefficients	95.0% Confidence Interval for B		*t*	*p*
	B	Std. Error	Beta	Lower Bound	Upper Bound		
Family member abroad	52.439	14.193	0.162	24.559	80.32	3.695	**0.000**
Marital status (single)	15.71	14.294	0.047	−12.368	43.788	1.099	>0.05
Financial situation (low)	5.484	15.842	0.015	−25.637	36.604	0.346	>0.05
Paying for studies	21.198	13.478	0.066	−5.277	47.673	1.573	>0.05
Residence (rural)	32.082	14.537	0.094	3.525	60.639	2.207	**0.028**
Gender (female)	1.405	25.916	0.002	−49.503	52.313	0.054	>0.05
Unsatisfied with potential income in Romania	54.702	13.785	0.17	27.624	81.78	3.968	**0.000**
One foreign language	26.658	23.603	0.051	−19.707	73.023	1.129	>0.05
Two foreign languages	−16.882	14.536	−0.053	−45.436	11.672	−1.161	>0.05
R^2^ (Adjusted R^2^)	0.81 (0.65)						
F	5.244						
*p*	0.000						

Bold font indicates statistical significance, *p* < 0.05.

**Table 10:** Linear regression analysis for predictors of pull factor score.

	Unstandardized Coefficients		Standardized Coefficients	95.0% Confidence Interval for B		*t*	*p*
	B	Std. Error	Beta	Lower Bound	Upper Bound		
Family member abroad	−30.978	9.381	−0.146	−49.406	**−12.551**	**−3.302**	**0.001**
Marital status (single)	11.49	9.46	0.052	−7.093	30.073	1.215	0.225
Financial situation (low)	34.439	10.47	0.142	**−13.871**	**55.007**	3.289	**0.001**
Paying for studies	2.177	8.916	0.01	−15.337	19.691	0.244	0.807
Residence (rural)	−18.295	9.613	−0.082	−37.179	0.59	−1.903	0.058
Gender (female)	38.104	17.12	0.094	4.475	71.734	2.226	**0.026**
Unsatisfied with potential income in Romania	−22.478	9.126	−0.107	−40.405	**−4.551**	**−2.463**	**0.014**
One foreign language	6.961	15.594	0.02	−23.673	37.595	0.446	0.656
Two foreign languages	−16.573	9.627	−0.079	−35.484	2.338	−1.721	0.086
R^2^ (Adjusted R^2^)	0.64 (0.49)						
F	4.1						
*p*	0.000						

Bold font indicates statistical significance, *p* < 0.05.

**Table 11:** The impact of Brexit on emigration intentions.

		November 2016	November 2017	November 2018	January 2021	November 2022
**Did Brexit influence your migration plans?**						
I don’t plan to leave	N	35	32	30	40	74
	%	43.2%	37.2%	35.7%	48.8%	59.2%
Yes	N	9	5	11	5	6
	%	11.1%	5.8%	13.1%	5.8%	4.8%
No	N	37	49	43	37	45
	%	45.7%	57.0%	51.2%	45.1%	36%
**How did Brexit influence your migrations plans?**						
I don’t plan to leave	N	35	32	30	40	74
	%	43.2%	37.2%	35.7%	48.8%	59.2%
It didn’t influence me, the UK was not my destination	N	20	26	28	19	21
	%	24.7%	30.2%	33.3%	23.2%	16.8%
I still want to work in the UK	N	21	23	18	21	28
	%	25.9%	26.7%	21.4%	25.6%	22.4%
I changed my destination country	N	5	5	8	2	2
	%	6.2%	5.8%	9.5%	2.4%	1.6%

## Data Availability

The data are available on request from the first author.
